# Zebularine Induces Long-Term Survival of Pancreatic Islet Allotransplants in Streptozotocin Treated Diabetic Rats

**DOI:** 10.1371/journal.pone.0071981

**Published:** 2013-08-26

**Authors:** Henrietta Nittby, Peter Ericsson, Karolina Förnvik, Susanne Strömblad, Linda Jansson, Zhongtian Xue, Gunnar Skagerberg, Bengt Widegren, Hans-Olov Sjögren, Leif G. Salford

**Affiliations:** Institute of Clinical Sciences, Department of Neurosurgery, the Rausing Laboratory, Lund University, Lund, Sweden; Children’s Hospital Boston/Harvard Medical School, United States of America

## Abstract

**Background:**

Coping with the immune rejection of allotransplants or autologous cells in patients with an active sensitization towards their autoantigens and autoimmunity presently necessitates life-long immune suppressive therapy acting on the immune system as a whole, which makes the patients vulnerable to infections and increases their risk of developing cancer. New technologies to induce antigen selective long-lasting immunosuppression or immune tolerance are therefore much needed.

**Methodology/Principal Findings:**

The DNA demethylating agent Zebularine, previously demonstrated to induce expression of the genes for the immunosuppressive enzymes indolamine-2,3-deoxygenase-1 (IDO1) and kynureninase of the kynurenine pathway, is tested for capacity to suppress rejection of allotransplants. Allogeneic pancreatic islets from Lewis rats were transplanted under the kidney capsule of Fischer rats previously made diabetic by a streptozotocin injection (40 mg/kg). One group was treated with Zebularine (225 mg/kg) daily for 14 days from day 6 or 8 after transplantation, and a control group received no further treatment. Survival of the transplants was monitored by blood sugar measurements. Rats, normoglycemic for 90 days after allografting, were subjected to transplant removal by nephrectomy to confirm whether normoglycemia was indeed due to a surviving insulin producing transplant, or alternatively was a result of recovery of pancreatic insulin production in some toxin-treated rats. Of 9 Zebularine treated rats, 4 were still normoglycemic after 90 days and became hyperglycemic after nephrectomy. The mean length of normoglycemia in the Zebularine group was 67±8 days as compared to 14±3 days in 9 controls. Seven rats (2 controls and 5 Zebularine treated) were normoglycemic at 90 days due to pancreatic recovery as demonstrated by failure of nephrectomy to induce hyperglycemia.

**Conclusions/Significance:**

Zebularine treatment in vivo induces a long-lasting suppression of the immune destruction of allogeneic pancreatic islets resulting in protection of allograft function for more than 10 weeks after end of treatment.

## Introduction

There is an increasing clinical need for replacing malfunctioning organs or cells producing key substances by transplantation from allogeneic donors or from the patient himself. Although donor matching can minimize the histocompatibility barrier in the former case [1], life-long immunosuppressive medication is at present required to protect against immune rejection of the transplant [2]. Even in the case of transplantation of syngeneic cells, e.g., after cells have been genetically engineered to produce insulin in a patient with type-1 diabetes, a similar long-term immunosuppressive treatment will be needed to protect the transplanted cells from destruction by immune responses to the auto-antigens that originally caused the disease [3]. Presently, coping with the immune reactivity in these and similar cases will necessitate life-long immune suppressive therapy acting on the immune system as a whole, which is known to make the patients vulnerable to infections and to increase their risk of developing cancer [4].

A dramatic improvement will come when it will be possible to induce a long-lasting, antigen selective immunosuppression or tolerance to the key antigens in question for each individual patient. Besides avoiding life-long therapy, the most important advantage will be that there will be no induction of general immune suppression with its accompanying sensitivity to infections and enhanced cancer risk.

Zebularine is a methyl transferase inhibitor that in dividing cells will be incorporated into the DNA as a deoxy base replacing the deoxycytidine. Since it is a cytidine analog, it will function as deoxycytidine in the DNA when being transcribed by the RNA polymerases [5]. When Zebularine is incorporated into the DNA, it will inhibit the DNA methylases [5]. However, all methylated cytidine analogs will not be demethylated [6]–[7]. In studies of the effect of of Zebularine on the antigenicity of tumor cells [8], we found that treatment with a moderately high dose of Zebularine (100 µM) increased the expression of the gene of the enzyme indolamine-2,3-deoxygenase-1 (IDO1) in rat colon cancer cells in vitro and drastically reduced their immunogenicity. This effect on immunogenicity could be inhibited by the competitive inhibitor 1-methyl-tryptophan, which indicates that the Zebularine-induced inhibition of the tumor immunogenicity was indeed caused mainly by IDO1. In addition, Zebularine has been shown to induce a strong expression of IDO1 in rat bone marrow-derived dendritic cells generated *ex vivo* (unpublished data).Although the process of demethylation is to some extent probably random, our subsequent microarray studies on normal, polyclonally stimulated human peripheral monocytes revealed that several of the genes in the tryptophan pathway were preferentially upregulated in the presence of Zebularine, especially IDO1 and kynureninase (own unpublished data). It is conceivable that in each cell type there might exist a hierarchy of genes that are more prone than others to be demethylated in this manner. Subsequently, we have demonstrated a strong synergism between Zebularine and interferon gamma (IFN-γ) in inducing prolonged IDO1expression in human monocytic THP-1 cells ([7], unpublished data).

On the basis of these results we propose to use Zebularine as a novel conditioning treatment of recipients of transplants and a therapy for patients with autoimmune diseases. IDO1 and kynureninase are key enzymes in the catabolism of the essential amino acid tryptophan. It is well established that IDO1 is one of the key molecules expressed by tolerogenic dendritic cells both in experimental animals and in humans [9]. The basis for the suppressive effect on T-cells has been shown to be the extreme sensitivity of T-cells to tryptophan starvation [10] and the apoptotic effect of the catabolites [11]. Recently, data were presented that strongly suggested that the IDO1 molecule, independent of its enzymatic effect, also can function as a signal transducer for transforming growth factor beta (TGF-β1), another key immunosuppressive molecule expressed by many cell types including tolerogenic dendritic cells [12].

Taking into account the vital role of IDO1 for immunological tolerance, and the findings that Zebularine treatment induces IDO1 expression, such treatment might be a powerful tool for preventing immunogenic reactions against transplanted tissues and controlling autoimmune reactions by turning these into a tolerogenic state. In the present study we take a first step to establish this novel mode of immunosuppression by demonstrating that Zebularine treatment in vivo is capable of suppressing a strong immune rejection response towards pancreatic islet allografts differing from the recipient with regard to portions of major histocompatibility antigens [13].

## Materials and Methods

### Ethics Statement

All animal procedures were performed according to the practices of the Swedish Board of Animal Research and were approved by the Animal Ethics Committee, Lund-Malmö, Sweden (Permit number: M310-10). All efforts were made to minimize suffering.

### Animals

Lewis rats, Fischer 344 rats and Wistar rats were obtained from Charles River Laboratories (Germany) and kept at a 12-h light and 12-h dark cycle in rat hutches (2–3 in each cage) with free access to standard rodent chow and tap water.

All surgery was performed under isoflurane inhalation anaesthesia. No antibiotics were used. Buprenorphine (Temgesic, Schering-Plough, NJ, USA) was administered intramuscularly (0.05 mg/kg) after islet transplantation and nephrectomy to alleviate postoperative pain. After finishing the experiments, the rats were sacrificed by opening of the thoracic cavity and emptying of the inferior vena cava from blood under isoflurane anesthesia.

### Zebularine Treatment

The cytidine analogue Zebularine was obtained from Berry&Associates (Dexter, MI, USA) and dissolved in PBS (50 mg/ml). Zebularine (225 mg/kg) was administered by intraperitoneal injection. The dose is atoxic and does not affect the general condition of the rats. The dose has been shown to induce an increased IDO1 expression in rat spleens (unpublished data).

Before starting the allotransplantation study a pilot study was performed to verify that the Zebularine dose chosen would induce increased IDO1 expression *in vivo*. In this study male Wistar rats received daily treatment with Zebularine (225 mg/kg, 2 rats) or PBS (controls, 2 rats) for 7 days. The rats were sacrificed the day after the last Zebularine injection (day 8) and the spleens isolated and frozen in liquid nitrogen for isolation of RNA.

### RNA Isolation

Pieces of frozen spleens were homogenized in liquid nitrogen using mortar and placed in TRIzol reagent (Invitrogen, Life Technologies, Paisley, UK), and then total RNA was extracted according to Invitrogen’s protocol. Quality and quantity of isolated RNA was measured by gel electrophoresis and spectrophotometer.

### Quantitative Real Time PCR Analysis

The qRT-PCR analyses were performed using the SuperScript III Platinum Two-Step qRT-PCR Kit with SYBR Green (Invitrogen, Life Technologies, Paisley, UK). A total of 1 µg total RNA was used in a 20 µl RT reaction using a mixture of polydT and random hexamers primers. The cDNA obtained was diluted to a total volume of 80 µl and stored at −20°C.

The primer sequences for the different genes were designed using Gene Fisher software support (G. Giegerich, F. Meyer, C. Schleier-macher, ISMB-96). The primers used for amplification of the rat IDO1 (rIdo1) gene were 5′-GTTCTTCGCATATATTTGTCCGG-3′ (forward) and 5′-CAGGGGGCAGTGCAGGCCA-3′ (reverse) according to a rat cDNA sequence with a PCR product 248 bp in size. For amplification of the endogenous reference gene hypoxanthine guanine phosphoribosyl transferase (HPRT) forward primer 5′-CAAGCTTGCTGGTGAAAAGGA-3′ and a reverse primer 5′-CACAAACATGATTCA-AATCCCTGA-3′, according to the cDNA sequence, were used. The qRT-PCR was performed in 20 µl reaction consisting of 2 µl diluted cDNA, 0,3 µM of each primer, 1×bovine serum albumin (50 µg/ml) and 10 µl Platinum SYBR Green qRT-PCR superMix-UDG. The amplification of rIDO1 and HPRT were carried out in a Light Cycler (Roche Molecular Biochemical). The amplification of rIDO1 was carried out with the following thermal profile: UDG incubation at 35°C for 2 min, then denaturation at 95°C for 5 min, followed by 45 cycles at 94°C for 6 s, 56°C for 10 s, and 72°C for 14 s. The amplification of rHPRT was carried out as follows: UDG incubation at 35°C for 2 min, then denaturation at 95°C for 5 min, followed by 45 cycles at 94°C for 6 s, 57°for 10 s, and 72°C for 14 s. After amplification a melting curve analysis was performed. The qRT-PCR experiments were run in triplicate.

Data analysis was performed with Light Cycler software version 3 (Roche Molecular Biochemicals). The threshold level was determined using the software, according to optimization of the baseline and the standard curve. Standards were obtained by amplification of a control DNA sample in a RT-PCR, using the same primers, reagents, and conditions optimized for the real-time analysis. The IDO1 expression levels were normalized with the expression levels of HPRT.

### Induction of Diabetes

Streptozotocin (STZ) was obtained from Sigma-Aldrich Sweden AB (Stockholm, Sweden) and dissolved in PBS. A single dose of STZ (Sigma-Aldrich Sweden AB, Stockholm, Sweden) was administered intraperitoneally to recipient rats to induce diabetes. Blood glucose was monitored using a Bayer Contour glucometer (Bayer AB, Diabetes Care, Solna, Stockholm). Before performing the transplantation study, the dose of STZ needed to establish a stable diabetic state was determined. At moderately high doses, 30–45 mg/kg i.p., the rats (16 rats in total) developed diabetes, with blood glucose levels >20 mmol/l. None of the animals with induced diabetes returned to euglycemia. Our results are in line with previous studies [14]–[15], where a STZ dose of 30–40 mg/kg has been shown to effectively induce diabetes in Fischer 344 rats at a stable and moderately severe level, with hyperglycemia, polydipsia, polyuria, polyphagia and muscle wasting leading to weight loss. Therefore, STZ doses of 40 mg/kg were chosen for the transplantation studies.

### Isolation of Rat Pancreatic Islets

Male Lewis (allotransplantation) or Fischer 344 (syngeneic transplantation) rats (10–14 weeks old) were used as donors. The method described by He et al. [16] was used, with some modification. The rats were anaesthetized using isoflurane inhalation. The abdominal cavity was cut open, and the animals were sacrificed. The papilla Vateri was located and clamped and 20 ml of Hank’s buffered saline solution (HBSS) (Sigma-Aldrich Sweden AB, Stockholm, Sweden) with Collagenase P (Roche Diagnostics, Mannheim, Germany), at a concentration of 0.7 mg/ml, was injected into the common bile duct. The inflated pancreas was resected and incubated at 37°C for 30 min in HBSS. The dissociated islets were purified by gradient centrifugation, using Histopaque (Sigma-Aldrich Sweden AB, Stockholm, Sweden). Handpicked islets were washed with HBSS, counted, and then incubated overnight in RPMI 1640 (Life Technologies) with 10% fetal bovine serum at 37°C in 5% CO_2_ and 95% air. For each transplantation, islets from one to two donors were used.

### Islet Transplantation

Female Fischer 344 rats made diabetic with a single dose of STZ (40 mg/kg) were used as recipients. The rats were 10–14 weeks old when included in the study, with a median weight of 172±13 g. All rats used as recipients had a blood glucose level of ≥20 mmol/l at transplantation. A small incision was made on the left flank and the left kidney was located. The kidney capsule was cut open and 500–1000 (as counted under light microscope) islets were injected into the subcapsular space using a Hamilton syringe. The opening of the kidney capsule was sealed with Surgicel fibrillar (Johnson&Johnson) company) and 4-0 sutures were used to close the peritoneum and cutaneous incision. Blood-glucose was subsequently monitored, until the rats became euglycemic (defined as blood-glucose <10 mmol/l).

#### Syngeneic Fischer 344 transplantation

Before starting the allotransplantation study a syngeneic Fischer 344 to Fischer 344 transplantation study was performed. This was to ascertain that the transplantation technique and long-term graft survival would be adequate in this model. In the syngeneic transplantation study, four female Fischer 344 rats made diabetic with a single dose of STZ (30 mg/kg) each received 500–900 islets from male Fischer 344 rats.

#### Lewis to Fischer 344 allotransplantation

25 female Fischer 344 rats, made diabetic with STZ 40 mg/kg, each received 500–1000 islets from male Lewis rats. The rats were divided into treatment (14 rats) or control (11 rats) groups. Successful transplantation was defined as return to euglycemia (blood-glucose <10 mmol/l) postoperatively. Control animals were left without further treatment while the rats in the treatment group received daily treatment for 14 days with Zebularine (225 mg/kg, i.p.), with start day 6–8 after the transplantation. All intraperitoneal injections were given under light isoflurane inhalation anesthesia.

After successful transplantation, the rats were followed with blood-glucose measurements at least twice weekly, until rejection of transplant occurred (defined as blood-glucose levels ≥10 mmol/l for two consecutive measurements separated by at least 24 hours) or until the end of the observation period (90 days). If the rats became diabetic before the end of the 90 day observation period, they were sacrificed and the left kidney was removed and fixed in 4% formaldehyde. If the rats were euglycemic after 90 days, they were subjected to nephrectomy to verify dependence on the transplant to control euglycemia. Nephrectomy was performed by opening of the old incision, ligating the renal vein and artery off and resecting the left kidney after which the peritoneum and cutaneous incision were closed with 4-0 sutures. The kidney was spared for histological examination. The animals were then followed until they developed hyperglycaemia (which in this study would be for maximally 4 days) and were then sacrificed. If the animals remained euglycemic after nephrectomy they were followed up to maximally day 100 and it was assumed that their own pancreatic function had been regained.

### Histopathology

After dissection, the relevant kidneys were immersion-fixed in 4% buffered formalin and stored in 70% ethyl-alcohol for up to two months before being embedded in paraffin.

The kidneys were serially sectioned at 5 micrometer in a plane perpendicular to the long axis of the organ.

Every tenth section of the kidneys was stained with Haematoxylin and Eosin and examined in the microscope; sections containing an obvious transplant or a distinctive lesion were further analyzed and adjacent sections were immunohistochemically processed for the visualization of cells containing insulin.

These sections were deparaffinized and treated in a microwave oven for retrieval of antigen before being incubated with a mouse monoclonal antibody against insulin (AM20632PU-N) (Acris Antibodies GmbH, Herford, Germany) at a dilution of 1∶200 for two hours and subsequently with a biotinylated secondary antibody and ABC reagent 30 minutes each, these two later steps were carried out using a ready-to use Vectastain ABC kit (Vector Laboratories, CA, USA) where after the antigen-antibody complex was visualized using the DAKO Liquid DAB Substrate-Chromogen System (DAKO, CA, USA) and the sections were stained with Haematoxylin and Eosin. The sections were thoroughly rinsed in PBS before each step of the immunohistochemical procedure.

The sections were analyzed by a an observer blinded to the experimental protocol using a Zeiss light-microscope utilizing 12.5× eyepieces and a 20× EC plan-neofluar objective giving a field of view of 0.6 mm^2^.

The number of immunopositive cells within the transplant was counted as the number of immunopositive cellular profiles with a visible nucleus observed within the one visual field exhibiting the largest number of cells (− = no cells; + = 1–10 cells; ++ = 11–20 cells; +++ = 20 cells).

### Statistics

2-sided Mann-Whitney test was applied, as well as Kaplan-Meier analysis, for analysis of survival. Pearson correlation analysis was used to compute the correlation coefficient.

## Results

### Systemic Treatment with Zebularine Induces IDO1 in Rat Spleens

**Figure 1:**
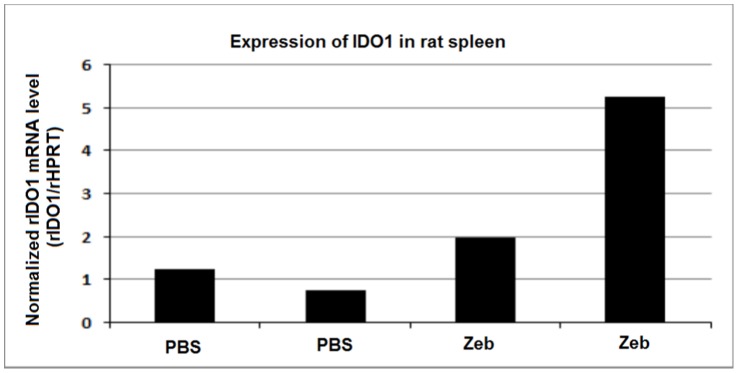
Expression of IDO1 in rat spleen. Analysis of the IDO1-gene expression in spleens from four Wistar rats treated with daily i.p. injections of Zebularine (225 mg/kg/day, two rats) or PBS (two rats) for 7 days. IDO1 expression was measured by Real-time qPCR fluorescence and the normalized rIDO1 expression level for each spleen sample calculated by rIDO1 expression divided by rHPRT expression. The normalized IDO1 expressions of spleens from Zebularine-treated rats are presented in relation to the mean of that of PBS-treated controls.

Systemic daily treatment of male Wistar rats with Zebularine (225 mg/kg) for 7 days induced a two- to fivefold increase in IDO1 expression in spleens, as compared to rats treated with PBS (Figure 1).

### Syngeneic Fischer-to-Fischer Transplantation

For syngeneic Fischer to Fischer transplantation, 500–900 islets cured the diabetic state induced by exposure to STZ. Removal of the islet transplant after more than 3 months of euglycemia by nephrectomy lead to hyperglycemia in 3 out of 4 cases. This was taken as proof that the induced normoglycemia in these 3 rats was dependent on the grafted islets. All 3 animals also had insulin-containing cells detectable under the kidney capsule. In the fourth case, graft removal did not lead to hyperglycemia, a recovery of pancreatic insulin production that is known to occur in a proportion of rats exposed to STZ [17]–[19]. In this animal insulin-containing cells were detected in the kidney. This illustrates the necessity to check whether a long-lasting normoglycemic state is dependent on transplant survival or recovery of pancreatic insulin production.

### Prolonged Survival of Pancreatic Islet Allografts in Rats Treated with Zebularine

**Figure 2:**
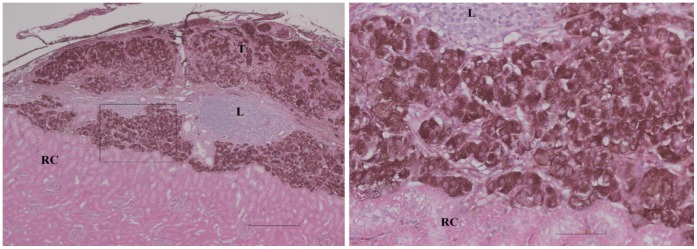
Survival of transplant in Zebularine treated rat at the end of the 90-days observation period. Left: Insulin positive cells (red-brown) of the transplant (T) are seen superficial to the normal renal cortex (RC) (semiquantitative scoring of +++). A large collection of leucocytes (L) is buried deep into the transplanted tissue. Bar is 400 µm. Right: The area outlined in a higher magnification; insulin positive cells are easy to distinguish from adjacent leucocytes and renal parenchyma. Bar is 80 µm.

**Figure 3:**
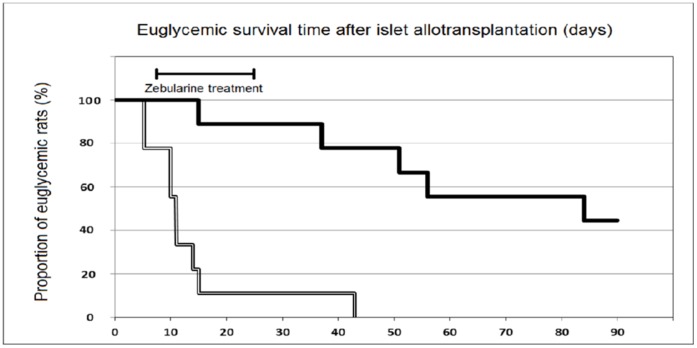
Euglycemic survival after allotransplantation. Days of euglycemia for animals which finally developed hyperglycemia (nine Zebularine treated animals out of which four were hyperglycemic after nephrectomy at day 90 versus nine control animals) (p<0.001, 2-sided Mann Whitney). Zebularine treated animals as black line, and control animals as white-and-black line.

**Figure 4:**
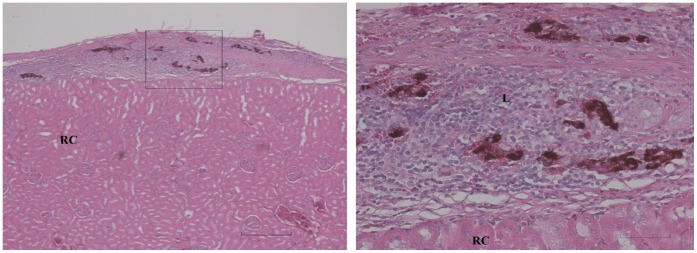
Kidney from Zebularine treated animal which became diabetic during the observation period at day 15. Left: Insulin positive cells within or beneath the kidney capsule (semiquntitative scoring of ++). Bar is 400 µm. Right: A higher magnification reveals small numbers of insulin positive cells surrounded by leucocytes (L) and connective tissue superficial to the renal cortex (RC). Bar is 80 µm.

Rats normoglycemic for 90 days after allografting were subjected to transplant removal by nephrectomy to confirm whether euglycemia was indeed due to a surviving insulin producing transplant, or alternatively was a result of recovery of pancreatic insulin production (see above). A total of 7 rats (2 controls and 5 Zebularine treated) were normoglycemic at day 90 due to pancreatic recovery as demonstrated by failure of nephrectomy to induce hyperglycemia and these rats were excluded from further evaluation. Of the remaining 9 Zebularine treated rats, 4 were still euglycemic after 90 days and became hyperglycemic after nephrectomy (histological scoring +++ for three of them (see Figure 2) and ++ for one). The mean length of time the Zebularine group stayed normoglycemic was 67±8 (SEM) days after transplantation as compared to 14±3 (SEM) days for the 9 controls (Figure 3). This difference is highly significant (p<0.001, 2-sided Mann-Whitney). Median length of euglycemia was 84 days in the Zebularine treated group and 11 days in the control group. In the five Zebularine treated animals, which developed hyperglycemia before the end of the 90 days observation period was reached indicating rejection of the allograft, although delayed, there were remaining insulin expressing cells in 2 animals (histological scoring of ++ for both of them, diabetic at days 15 and 37) (Figure 4) but in 3 there were no such cells detectable (diabetic at days 51, 56 and 84).

**Figure 5:**
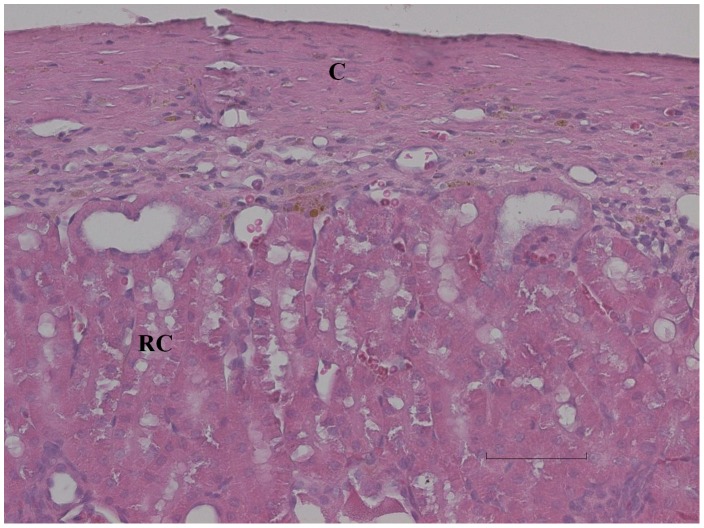
No viable transplant in control animal. Control animal which became diabetic at day 14 after transplantation, where no insulin positive cells are seen within the thickened renal capsule (C) (semiquantitative scoring of 0). Bar is 80 µm.

**Figure 6:**
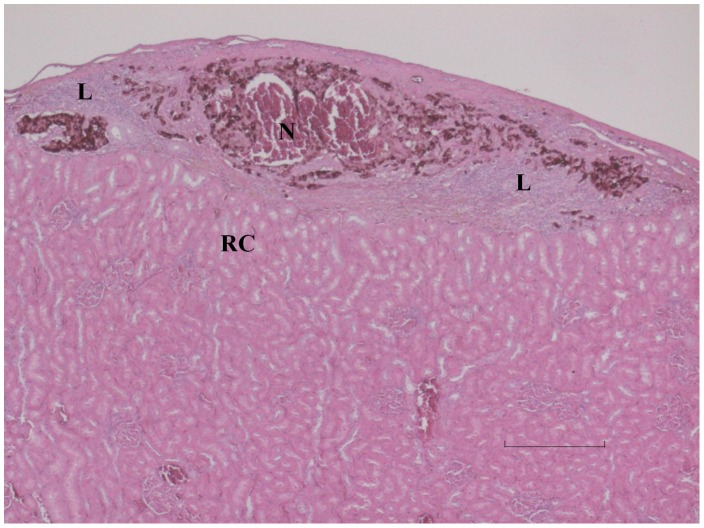
Kidney from control animal which became diabetic day 43 after transplantation. Under the kidney capsule is a relatively high amount of insulin positive cells (semiquantitatively scored to +++), yet not enough to keep the animal euglycemic. N denotes necrotic central area of the transplant and L accumulations of leucocytes. RC is normal renal cortex. Bar is 400 µm.

Nine control animals developed hyperglycemia during the observation period. Eight of these were analyzed. Six of the animals had no insulin positive staining in their kidneys (Figure 5), whereas two rats had remaining insulin expressing cells in the kidney scoring as ++ or +++ (diabetic first at day 43 after transplantation, Figure 6).

There was no significant correlation between the amount of transplanted islets, and survival (Pearson coefficient −0.28 for the controls and −0.35 for the Zebularine treated animals, p-value n.s.).

## Discussion

In this study, we show that the DNA demethylating agent Zebularine has a strong suppressive effect on the rejection response against Lewis rat pancreatic islets allotransplanted under the kidney capsule of diabetic Fischer rats. The survival of the transplants was monitored by frequent blood sugar measurements and a confirmed value >10.0 was regarded as the time point when the transplanted islets had been rejected and the insulin levels were no longer sufficient to maintain normoglycemia. Rats that were still normoglycemic at 90 days after allografting were nephrectomized to determine whether their normal blood sugar was a result of the survival of the transplant, or was instead due to the recovery of insulin production in the pancreas. Such a recovery is to be expected in a proportion of rats after exposure to Streptozotocin [17]–[19]. Animals being normoglycemic after nephrectomy were excluded from evaluation since the survival of their islet allograft could not be adequately determined. The mean survival time of the allograft as judged in this way was highly significantly prolonged in the Zebularine treated group compared to the controls receiving no therapy after the allotransplantation (mean of 67±8 days *vs* 14±3 days, p<0.001). Out of 9 Zebularine treated rats maintaining their grafts for 90 days without developing hyperglycemia, 4 were shown by nephrectomy to still require their allografted islets to maintain their normoglycemic state. The other 5 rats remained normoglycemic upon graft removal and were consequently excluded from evaluation because of recovery of the pancreatic insulin production.

Kidneys which had received transplants of allogeneic pancreatic islets were investigated immunohistologically for the presence of cells expressing insulin. As expected, all the 4 Zebularine treated rats that remained normoglycemic for 90 days were found to have insulin expressing cells in the kidney. Among the 5 Zebularine treated rats that became hyperglycemic within 90 days, 2 also had remaining insulin expressing cells in their kidneys, although obviously not sufficiently many to maintain normoglycemia. No insulin containing cells could be detected in the other 3 rats. Eight of the 9 controls were also investigated and while 6 had no detectable insulin expressing cells, 2 had remaining insulin containing cells, although obviously not sufficient in quantity to maintain a normoglycemic state.

Based on our previous analyses, the mechanism of action of this novel mode of immunosuppressive treatment with Zebularine is the induction of expression of the rate limiting tryptophan catabolizing enzyme IDO1. This occurs preferentially in certain subsets of dendritic cells, initiating the kynurenine pathway. The dose of Zebularine used in the present study was chosen on the basis of our previous finding that daily treatment with Zebularine (225 mg/kg, i.p.) for three weeks induces a significantly enhanced expression of IDO1 in the spleen as demonstrated by PCR (unpublished data). In the present study, this was verified in healthy control rats before starting the allotransplantation study. Here, daily treatment with Zebularine (225 mg/kg, i.p.) for 7 days induced a two- to fivefold increase in IDO1 expression in the spleen (Figure 1).

In at least some cells, including dendritic cells, Zebularine activates expression of IDO1 and kynureninase. These enzymes have been reported to suppress T lymphocytes by creating local tryptophan shortage [10] and excess concentrations of tryptophan catabolites [11]. Among cytokines IFN-γ is the strongest inducer, although in vitro the effect disappears rapidly after removal of the cytokine to return to the base level within approximately 24 h (unpublished data). We have demonstrated a very strong synergistic induction of IDO1 expression by Zebularine and IFN-γ in the human monocytic cell line THP-1 in vitro (unpublished data). This synergistic effect is also long-lasting and is observed more than a week after removal of the IFN-γ. This is of particular interest since IFN-γ is commonly expressed in areas of graft rejection and inflammatory sites in patients with autoimmunity, in which Zebularine might consequently be expected to induce a synergistic, particularly strong IDO1 expression. In addition to its enzymatic action to catabolize tryptophan, it was recently reported that the IDO1 molecule can also function as a signal transducer for TGF-β1 [12], enabling this cytokine to activate the alternative NFκB pathway. Through this pathway TGF-β1 may induce its own production and the expression of IDO1 as well. This might have the potential of creating a long-lasting immunosuppressive loop through the generation of regulatory T lymphocytes via the effect of TGF-β1 and T-cell inhibition via the IDO1.

An analogously suppressed allorejection has previously been reported after IDO1 gene transfer into the tissue to be grafted [20] and this has been interpreted to be due to a T-cell inhibitory effect of the IDO1. Furthermore, a similar transfer of the IDO1 gene into bone marrow derived dendritic cells and subsequent use of these cells to treat mice with collagen induced rheumatic arthritis (RA) has been reported to strongly suppress the inflammatory disease process in this murine RA model [21]–[22]. At the present time, however, an increased risk of cancer development by this type of gene transfer and gene manipulation cannot be ruled out, why they are mainly reserved for life-threatening diseases.

The results of our present investigation are important, representing the first demonstration that systemic administration of the IDO1- and kynureninase-inducing drug Zebularine is causing a long-lasting suppression of allograft rejection as a sign of immunosuppression and possibly immune tolerance. The results indicate that Zebularine might provide a tool by which a short period of drug therapy enables the permanent survival of allografted cells and organs, such as pancreatic islets. A similar protection against autoimmune destruction might be anticipated for autologously grafted, insulin-producing cells differentiated from stem cells, when the technology to produce them has been sufficiently developed. The mode of using Zebularine, alone or in combination with other modulators of immune responsiveness, is to be further explored both technically and in relation to application on autoimmunity. This will include expansion of the work on using dendritic or other cells after in vitro exposure of these cells to Zebularine, relevant antigens, and/or other immune cells as a mean to enhance antigen selectivity, efficiency and safety.
